# Full-scale structural model of the Inferior Olive and Olivocerebellar projection constructed from constraining meshes and directed growth

**DOI:** 10.1186/1471-2202-14-S1-P190

**Published:** 2013-07-08

**Authors:** James Kozloski, Viatcheslav Gurev

**Affiliations:** 1 Computational Biology Center, IBM T.J. Watson Research Center, Yorktown Heights, NY, USA

## 

Constructing a full scale model of a brain structure demands several considerations. First, to be useful, the model must be of a computable size. Previously, we mapped the computation of dense multicompartmental models of neural tissue to the Blue Gene/P supercomputer at ~250 neurons per node of this machine. A ~30k neuron structure such as the Inferior Olive [1] is therefore well within the limits of today's machines, and so we aimed to build a structural model of this structure that could appropriately constrain a hypothetical physiological model.

**Figure 1:**
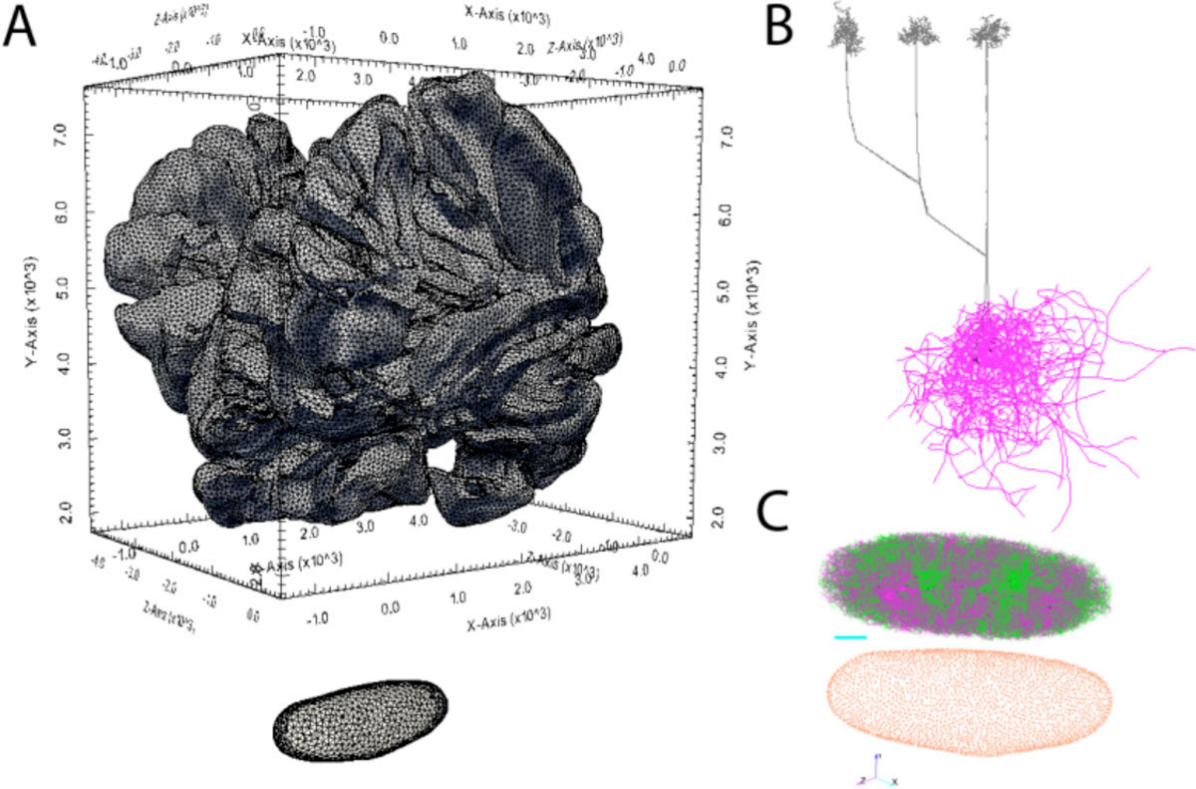
**A. Bounding triangular meshes for cerebellar granule cell layer (top) and inferior olivary nucleus (bottom), embedded in brain coordinates**. **B**. Granule cell layer mesh constrains waypoint-based axonal growth into the Purkinje cell layer of olivary neurons, where they ramify according the Purkinje cell self-referential growth model. **C**. Olivary nucleus mesh constrains placement of cell bodies and repels dendrites as they grow according to our model of self-referential forces, resulting in characteristic spirals.

Next, to constrain olivary physiology appropriately with our structural model, we included axons in addition to the usual structural constraints on neuronal integration imposed by dendritic morphologies, because spike generation and bursting properties of olivary neurons depend on axonal length [2]. To create our structural model, we first constructed a 3-D mesh from a segmented stack of slices through the granule cell and Purkinje cell layers of the rat cerebellum. Through this mesh, we required olivocerebellar projections and climbing fibers to course (Figure 1A, 1B) to arrive at and create Purkinje cell ramifications [3]. This method generates our distribution of axon lengths.

Finally, we constrained dendritic morphology using a version of our previous model of self-referentially biased dendritic growth [4], modified to give a better match to olivary neuron morphology. This new model takes as input a 3-D mesh, which approximates nucleus boundaries using measurements of the Inferior Olive size and composition [1]. As before, we generated dendritic arbors to match morphological measures [5], but determined that their characteristic spiral shape depends on a repulsive force from the bounding mesh. The full 24,815 dendritic arbors were then generated on 64 nodes of Blue Gene/Q, running 8 tasks/node, and 8 threads/task in <15 mins. (Figure 1C). We discuss future physiological models, including gap junctions, constrained by this structural model.
